# Surfactant-Enhanced Solubilization of Chlorinated Organic Compounds Contained in DNAPL from Lindane Waste: Effect of Surfactant Type and pH

**DOI:** 10.3390/ijerph17124494

**Published:** 2020-06-23

**Authors:** Raúl García-Cervilla, Arturo Romero, Aurora Santos, David Lorenzo

**Affiliations:** Chemical and Materials Engineering Department, University Complutense of Madrid, 28040 Madrid, Spain; raugar05@ucm.es (R.G.-C.); aromeros@quim.ucm.es (A.R.); aursan@quim.ucm.es (A.S.)

**Keywords:** DNAPL, surfactants, partition, lindane wastes, chlorinated organic compounds

## Abstract

Application of surfactants in the remediation of polluted sites with dense nonaqueous phase liquid (DNAPL) still requires knowledge of partitioning between surfactants and pollutants in the organic and aqueous phases and the time necessary to reach this balance. Two real DNAPLs, generated as wastes in the lindane production and taken from the polluted sites from Sabiñanigo (Spain), were used for investigating the solubilization of 28 chlorinated organic compounds (COCs) applying aqueous surfactant solutions of three nonionic surfactants (E-Mulse^®^ 3 (E3), Tween^®^80 (T80), and a mixture of Tween^®^80-Span^®^80 (TS80)) and an anionic surfactant (sodium dodecyl sulfate (SDS)). The initial concentrations of surfactants were tested within the range of 3–17 g·L^−1^. The pH was also modified from 7 to >12. The uptake of nonionic surfactants into the organic phase was higher than the anionic surfactants. Solubilization of COCs with the nonionic surfactants showed similar molar solubilization ratios (MSR = 4.33 mmol*_COCs_·*g^−1^*_surf_*), higher than SDS (MSR = 0.70 mmol*_COCs_·*g^−1^*_SDS_*). Furthermore, under strong alkaline conditions, the MSR value of the nonionic surfactants was unchanged, and the MSR of SDS value increased (MSR = 1.32 mmol*_COCs_·*g^−1^*_SDS_*). The nonionic surfactants did not produce preferential solubilization of COCs; meanwhile, SDS preferentially dissolved the more polar compounds in DNAPL. The time required to reach phase equilibrium was between 24 and 48 h, and this contact time should be assured to optimize the effect of the surfactant injected on COC solubilization.

## 1. Introduction

Contamination of soil and groundwater by organic compounds from industrial activities has become a major environmental problem [1]. This contamination is often due to the accidental release or intentional dumping of hydrophobic organic liquid phases into the environment, resulting in a separate liquid phase, referred to as nonaqueous phase liquids (or NAPLs), that persists in the subsurface [2]. Nonaqueous Phase Liquids are hydrophobic organic phases that show properties and behavior other than dissolving contaminant plumes. NAPLs whose density is lower than that of water are light NAPLs (LNAPLs) [3,4], and NAPLs with a higher density than water are called dense nonaqueous phase liquids (DNAPL).

DNAPLs include common industrial solvents (trichloroethylene, perchloroethylene, carbon tetrachloride, dichloromethane) or other hazardous substances such as creosote and coal tar. Other chlorinated organic pollutants forming DNAPLs include pesticides and chlorinated compounds used for their synthesis [5,6,7]. Most of these DNAPLs are persistent in the environment due to their hydrophobic nature and low biodegradability, characterized by high toxicity and bioaccumulation and, in some cases, carcinogenesis [8]. DNAPLs can migrate by density through the subsurface to greater depths, and a significant mass of DNAPL can be trapped in the soil pores [9,10] or soil fractures [5,11].

The remediation of sites polluted by DNAPLs poses essential technical and economic challenges. Conventional treatment technologies such as pump and treat have potentially high life cycle costs [12,13], and a feasible solution to improve the solubilization and mobilization of these DNAPLs is the Soil Flushing treatment where an aqueous solution containing the surfactant (with other possible amendments) is injected into the subsurface and then extracted and treated on-site [14,15,16,17,18,19,20,21,22]. 

Surfactants are amphiphilic compounds, and the hydrophilic group is an ionic (cationic, anionic) or highly polar group (nonionic) [23]. When a surfactant is added to the aqueous phase, surface tension decreases as surfactant concentration increases until the critical micelle concentration (CMC) is reached. At this point, tension remains constant as more and more surfactant is added to the solution [24]. However, although surface tension remains constant, the concentration of solubilized chlorinated organic compounds (COCs) increases when the concentration of surfactants in the solution increases [25]. 

Therefore, the equilibrium of the compounds presented in the DNAPL between the aqueous and the organic phase must be known for a reliable design of the Soil Flushing remediation treatment. This equilibrium is modeled using the mass solubilization ratio (WSR) or molar solubilization ratio (MSR). WSR is the ratio of the mass of solubilized organic compounds per unit of surfactant mass in micellar solution. MSR is defined as the moles of solubilized organic compounds per mole of surfactant in micellar solution [26,27]. Sharmin et al. calculated the MSR for perchloroethylene with Triton X-100 as a surfactant obtaining a value of 2.1 mol*_j_·*mol^−1^*_surf_* [28]. Kibbey et al. reported values of MSR in the range of 3.99–17.82 mol*_j_·*mol^−1^*_surf_* depending on the volume ratio of organic and aqueous phases using tetrachloroethylene and Tergitol NP15 as surfactants [29]. Zimmerman et al. reported apparent MSR values for different compounds (trichloroethylene, tetrachloroethylene, chlorobenzene, 1,2-dichlorobenzene) and by using different nonionic surfactants in the range of 0.5 to 4 mol*_j_·*mol^−1^*_surf_* [30]. Kang et al. also provided values of MSR for Tween 80, 40, and 20 using trichloroethylene and tetrachloroethylene as organic compounds. They found values around 8.3 mol*_j_·*mol^−1^*_surf_* for the Tween 40/trichloroethylene system and around 5 mol*_j_·*mol^−1^*_surf_* for Tween 80/tetrachloroethylene [31]. 

The extent of micellar solubilization depends on many factors (surfactant structure, aggregation number, micelle geometry, hydrophilic/lipophilic balance (HLB), ionic strength, temperature, and the size and chemistry of the solute) [32]. The HLB value is one of the most investigated parameters, but this number cannot confirm the required concentration of the emulsifying agent or the stability of the emulsions [23]. Moreover, there is a lack of studies carried out using real mixtures of organic compounds [33].

Additionally, due to the amphiphilic nature of the surfactant molecule, when a surfactant solution is in contact with an organic phase (such as the DNAPL), the partition of the surfactant between aqueous and organic phases has to be considered to determine the remaining amount of surfactant in solution [30,31]. The partition of surfactant between phases can reduce their active concentrations in an aqueous phase for the solubilization and mobilization of pollutants [31,34], affecting the effectiveness of the remediation treatment. The partition of the surfactant between both phases has been scarcely studied in literature [31,32,35]. Moreover, in these studies, the organic phase, as a DNAPL model, was composed of a single compound (chlorobenzene, 1,2-dichlorobenzene, 1,1,2-trichloroethane [36], perchloroethylene [28], tetrachloroethylene [29,30,36], dichloromethane, chloroform [30]). Only Yang et al. [37] studied binary mixtures of trichloroethylene and perchloroethylene. From literature results, it seems that nonionic surfactants present a higher affinity for the organic phases than anionic surfactants [31].

Correlations between the partition of a surfactant in both phases and the properties of that surfactant have been investigated in the literature. Catanoiu et al. [38] used three commercial nonionic surfactants (polyoxyethylene alkyl ethers, alkyl dimethyl phosphine oxides, and alkyl glycosides) with different water-in-alkane systems. They found that the affinity of the surfactant for the organic phase increases by increasing the surfactant alkyl chain length. Cowell et al. [36] studied the partitioning of ethoxylated nonionic surfactants in pure organic compounds (chlorobenzene, 1,2-dichlorobenzene, tetrachloroethylene, 1,1,2-trichloroethane), reporting that the concentration of the surfactant in the organic phase decreases when the polarity of this organic phase increases. This conclusion was also supported by the results of Kang et al., who studied the partitioning of different nonionic surfactants using pure compounds as an organic phase [31]. As commented before, the partitioning of surfactants between organic and aqueous phases using real DNAPL mixtures is not available in literature.

The pH effect on the partitioning equilibria has been scarcely studied, although it is proved that alkaline conditions can produce dehydrochlorination reactions of pesticides as lindane [39,40] or alkaline hydrolysis of organophosphorus pesticides, such as parathion and methyl parathion [33], generating less toxic products. Muff et al. [33] studied the solubilization of a NAPL compounded of two organophosphorus pesticides (50% w:w) in a polluted soil under strong alkaline conditions using several surfactants.

In this research, the partitioning equilibrium of surfactants and organic compounds between the organic and the aqueous phases has been studied using two different DNAPL samples. These DNAPLs were extracted from Sardas (S) and Bailin (B) landfills, caused by the dumping of liquid wastes from lindane production [7] containing up to 28 different chlorinated organic compounds [6]. The residues of the lindane produced by the company INQUINOSA were first disposed in the Sardas landfill, and later, in the Bailin landfill (Sabiñanigo, Spain), both unlined. The liquid waste dumped (DNAPL) had migrated by density forces through the subsoil in both landfills (Sardas and Bailin), with the corresponding conceptual models available in the literature [7,41]. The detection of this liquid waste was found at very variable depths, from 40 m deep under the ground level to the surface [7]. The groundwater in both landfills is connected to the Gallego river, and the solubilization of DNAPL in groundwater is a significant risk for the nearby river and reservoir [7,41].

To the best of our knowledge, the works available in literature studying these partitioning equilibria use pure organic compounds [30,31,36] or binary mixtures [42] at neutral pH. 

In addition, the effect of alkaline pH on partition equilibria (scarcely studied in literature) is analyzed since strong alkaline conditions can be used in a real treatment to promote the dehydrochlorination reactions of the most toxic COCs in DNAPLs.

Biodegradable nonionic and ionic surfactants have been selected, while good biodegradability is required for the application of a surfactant in aquifer remediation. Tween 80 and Span 80 or their mixtures have been proved to be readily biodegradable [43,44] and will be studied in this research. A commercial surfactant, E-Mulse^®^ 3, used in the remediation of sites with DNAPLs at full scale [22] has also been tested. Finally, sodium dodecyl sulfate (SDS), a widely used surfactant in soil remediation studies [45], has been selected as an anionic surfactant. 

## 2. Materials and Methods 

### 2.1. Chemicals

Three nonionic surfactants and an ionic surfactant were used. The nonionic surfactants tested were E-Mulse^®^ 3 (E3), Tween^®^80 (T80), and a mixture of Tween^®^80 at 35% and Span^®^80 at 65% (TS80). This mixture was tested in a previous laboratory study carried out by the Aragon Government using a DNAPL from the Sardas landfill (Sabiñanigo, Spain) [46]. Moreover, the three nonionic surfactants were biodegradable and non-toxic.

The anionic surfactant selected was sodium dodecyl sulfate (SDS), which is also typically used in soil washing. The identification of surfactants and their main chemical properties are shown in the Supplementary Material, Table S1. As can be seen, a nonionic surfactant presents a lower micellar concentration (CMC) than SDS.

Two different DNAPL samples obtained from the Sabiñánigo landfills and provided by the company EMGRISA and the Aragon Government were used. One DNAPL sample was obtained from the Sardas landfill (S) and the other sample from the Bailin landfill (B). The composition (molar fraction) of DNAPLs used in this research is summarized in Table S2 of the Supplementary Information. In this table, the average molecular weight MW_DNAPL_ for both DNAPLs is also included (232 mg·mmol^−1^ and 191 mg·mmol^−1^ for DNAPL: B and S, respectively). As can be seen, both DNAPLs contain up to 28 chlorinated organic compounds (COCs): chlorobenzene (CB) and different isomers of dichlorobenzene (DCBs), trichlorobemzenes (TCBs), tetrachlorobenzene (TetraCBs), pentachlorocyclohexene (PentaCXs), hexachlorocyclohexanes (HCHs), and heptachlorocyclohexanes (HeptaCHs). Compound distribution is quite similar to that mentioned in previous research [6]. These COCs represent more than 95% of the DNAPL mass.

### 2.2. Solubility Experiments

Solubility experiments were conducted in sealed GC 20 mL glass vials without headspace, closed with PTFE caps. A mass of 0.4 g of each organic phase, DNAPL: B, and DNAPL: S, was added to 19.6 g of the aqueous phase containing the surfactant (surfactant concentration ranging from 0 to 17 g L^−1^). These experiments were carried out on both neutral and alkaline pH (pH > 12). An alkaline pH was achieved by adding NaOH up to 7 g L^−1^ in the aqueous phase containing the surfactant. Table S3 provides a summary of the conditions of the experiments carried out. Six identical vials, which were sacrificed at different times of agitation, were prepared for each test.

The biphasic mixture (organic and aqueous) was agitated using a magnetic stirrer under room conditions for 5 h at room-controlled temperature (T = 22 ± 2 °C). Then, the agitation was stopped and different vials were sacrificed at 0.5, 1, 3, 24, 48, and 75 h to analyze the aqueous emulsion. The experiments were carried in triplicate. The differences were lower than 10%. 

### 2.3. Analytical Methods

The qualitative identification of dissolved COCs in the aqueous phase was carried out using a gas chromatograph (GC) (Agilent 6890 N, Sante Clara, CA, USA) along with a mass selective detector (Agilent MSD 5975B, Sante Clara, CA, USA). The quantification of COCs in the emulsion was carried out using a GC (Agilent 6890, Sante Clara, CA, USA) with both a flame ionization detector (FID) (Sante Clara, CA, USA) and an electron capture detector (ECD) (Sante Clara, CA, USA), simultaneously. The GC methods are described elsewhere [6]. The aqueous phase samples containing surfactants were previously diluted 1:10 with methanol to analyze the COCs.

The total organic carbon (TOC) of the supernatant (aqueous phase with the surfactant and solubilized COCs) was measured once the equilibrium was reached between phases (COC concentration in emulsion did not change over time). A Shimadzu TOC-V (Kyoto, Japan) analyzer was used. The concentration of nonionic surfactant under equilibrium conditions was determined from the TOC value and sum of COCs in the emulsion.

Ionic surfactant (SDS) concentration in aqueous phases was quantified by measuring sulfate anions in an aqueous solution using IC (Metrohm 761 Compact IC, Herisau, Switzerland). The column used as a stationary phase was Metrosep A SUPP5 5-250 (250 mm long, 4 mm wide) (Gallen, Switzerland), and the mobile phase used was an aqueous solution of Na_2_CO_3_ (3.2 mmol·L^−1^) and NaHCO_3_ (1 mmol·L^−1^).

To ensure that pH conditions were maintained throughout the entire experiment, the pH was also analyzed in the samples using a 914 pH/Conductometer (Metrohm, Herisau, Switzerland).

## 3. Results and Discussions

### 3.1. Partitioning of a Surfactant under Equilibrium Conditions

When equilibrium was reached, the concentration of surfactant absorbed in the organic phase *(C_surf,ORG_)_eq_* and the aqueous phase *(C_surf,AQ_)_eq_* were calculated from the surfactant mass balance as shown in the Supplementary Information. The mass of organic phase not dissolved at equilibrium conditions *(w_org_)_EQ_* was obtained from the DNAPL mass balance, as shown in equation (S1) to (S3) in the Supplementary Information. It was found that equilibrium conditions for the concentration of COCs in solution were obtained in under 24 h, remaining almost constant in the range of the 24–75 h time interval studied here.

**Figure 1:**
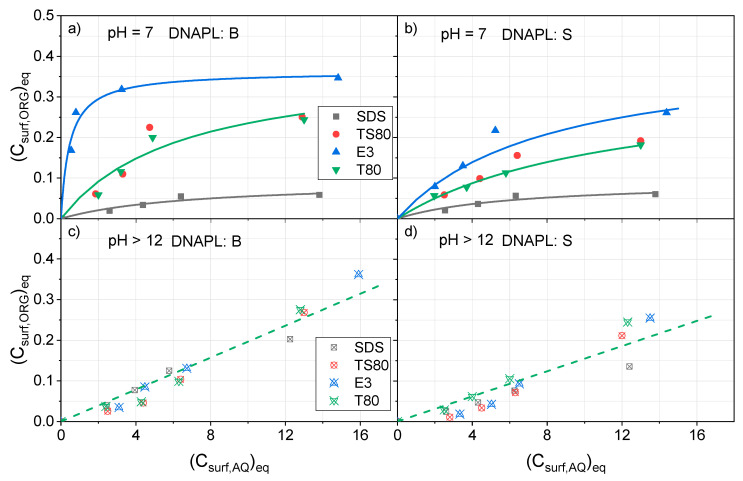
Partitioning of surfactants in organic-aqueous phases at (**a**) pH = 7, dense nonaqueous phase liquid (DNAPL): Bailin (B), (**b**) pH = 7 DNAPL: Sardas (S) (**c**) pH > 12 DNAPL: B (**d**) pH > 12 DNAPL: S *(C_surf,ORG_)_eq_* in g*_surf_*g*_ORG_*^−1^ and *(C_surf,AQ_)_eq_*, in g*_surf_*L^−1^. Experimental values as symbols and predicted values by Equation (1) or Equation (2) with parameters in Table 1 as lines.

The partition of surfactant between organic and aqueous phases for both DNAPLs (B and S) under equilibrium conditions is plotted at pH = 7 in Figure 1a,b and at pH > 12 in Figure 1c,d. (Data obtained after 48 h are used).

As can be seen in Figure 1a, the concentration of E3 in organic phase B at neutral pH reaches an asymptotic value, about 0.35 g*_surf_·*g^−1^*_ORG_*, when surfactant concentration in the aqueous phase is higher than 4 g·L^−1^. Lower partition ratios between the organic and aqueous phases were obtained using T80, TS80, and SDS. The partition ratios of T80 and TS80 were similar. Meanwhile, the lowest values were found with SDS. Moreover, asymptotic values are not achieved with T80, TS80, and SDS for concentrations lower than 15 g·L^−1^. Differences in the partition behavior of the surfactant tested at neutral pH indicate a higher affinity of E3 for organic phase B.

By comparing the results in Figure 1a,b, it can be deduced that surfactants have a lower affinity for DNAPL: S than for DNAPL: B. The highest discrepancies are found for the nonionic surfactant. This fact can be explained by differences in the composition of each organic phase B and S. It has been described in literature that the lower the polarity of the organic compounds, the higher the absorption of the nonionic surfactant in the organic phase [36]. As can be seen in Table S2, molar fractions of PentaCX, HexaCX, HCH, and HeptaCH isomers are higher in DNAPL: B than in DNAPL: S. On the contrary, molar fractions of DCB, TCB, and TetraCB isomers are higher in DNAPL: B. Therefore, the polarity of DNAPL: B is expected to be higher and, consequently, the affinity of the nonionic surfactant lower [36].

Results obtained at pH > 12 are shown in Figure 1c,d for DNAPL: B and S, respectively. As can be seen, the partition behavior of the four surfactants is similar for each DNAPL, and the differences found can be due to experimental error. Moreover, almost linear relationships are found between surfactant concentration in the organic and aqueous phases, and asymptotic values are not noticed in the concentration range studied. Surfactant concentration in the organic phase is slightly lower in DNAPL: S than in DNAPL: B. It can be generally noted that the affinity of the nonionic surfactants for the organic phase is more inferior in alkaline conditions than at neutral pH, at least when the surfactant concentration in the aqueous phase is lower than 15 g·L^−1^. On the contrary, the higher the pH, the higher the affinity of SDS for the organic phase. These effects can be explained, taking into account that the presence of electrolytes in the aqueous phase can modify the partition of the surfactant [34]. Moreover, alkaline conditions can alter both the polarity of the DNAPL surface and the surfactant micelles, thus affecting the surfactant partition.

To model the partition of surfactant between organic and aqueous phases, some authors [30,31,37] have used Langmuir isotherms, as shown in Equation (1).

*(C_surf,ORG_)_eq_ = [C_s,ORG_·K_s_·(C_surf,AQ_)_eq_]/(1 + Ks·(C_surf,AQ_)_eq_)*, (1)

where *C_s,ORG_* is the saturation concentration of surfactant in the organic phase, in g*_surf_·*g^−1^*_ORG_*, and *K_s_* the constant affinity of the surfactant for the organic phase, in L·g^−1^*_surf_*.

The experimental values shown in Figure 1a,b were fitted to Equation (1) by using non-linear regression, and the corresponding parameters calculated are shown in Table 1. Taking into account that similar behavior was obtained with surfactants T80 and TS80 (Figure 1a,b) and the differences can be explained by uncertainty in the experimental data, the results obtained with both surfactants were joined together. The statistical significance of parameters is summarized in Table S4. On the other hand, in Figure 1c,d, the partition of the surfactants between the organic and the aqueous phase presents a linear trend at pH > 12 and the concentrations applied. In this case, the expression in Equation (2) was proposed:

*(C_surf,ORG_)_eq_ = K_L_·(C_surf,AQ_)_eq_*. (2)

*K_L_* is the linear partitioning parameter [31] in g*_surf,ORG_·*L·g^−1^*_surf·_*g^−1^*_ORG_*. Equation (1) can be simplified using Equation (2) for small values of *K_L_*. The latter parameter is the product of *C_s,ORG_* by *K_s_* in Equation (2).

The experimental data obtained in alkaline conditions was fitted to Equation (2) using linear regression and a *K_L_* parameter was estimated. Since the four surfactants had a similar partitioning behavior under alkaline conditions, the experimental data was fitted together. A unique value of *K_L_* was obtained for each DNAPL, which is also shown in Table 1. As can be seen in Table 1, slightly lower values of *K_L_* were obtained with DNAPL: S, indicating that a lower affinity of this organic phase S for the surfactant is also found at an alkaline pH.

The corresponding values of *K_L_* at pH = 7 are also shown in Table 1. As can be seen, higher *K_L_* values for nonionic surfactants are obtained at a neutral pH, for both DANPLs B and S. On the contrary, higher *K_L_* values are obtained using the anionic surfactant SDS under alkaline conditions. In all cases, it is confirmed that pH has a strong influence on the affinity of the surfactant for the organic phases.

### 3.2. Solubilization of COCs

The solubilization experiments carried out are summarized in Table S3. In Figures S1–S5 of the Supplementary Material, some photos of the emulsions before agitation (up), after stirring (centrum), and 75 h after the agitation stopped are shown. As can be seen, the emulsions had a light brown appearance after agitation, being darker in the experiments with DNAPL: S. The brown color settled overtime after the agitation stopped, due to the presence of some clay interbedded in both DNAPL: B and S (more evident in DNAPL: S). As the total amount of COCs quantified represents more than 95% of the mass in both DNAPLs, the percentage of clay is lower than 5% in all cases.

**Figure 2:**
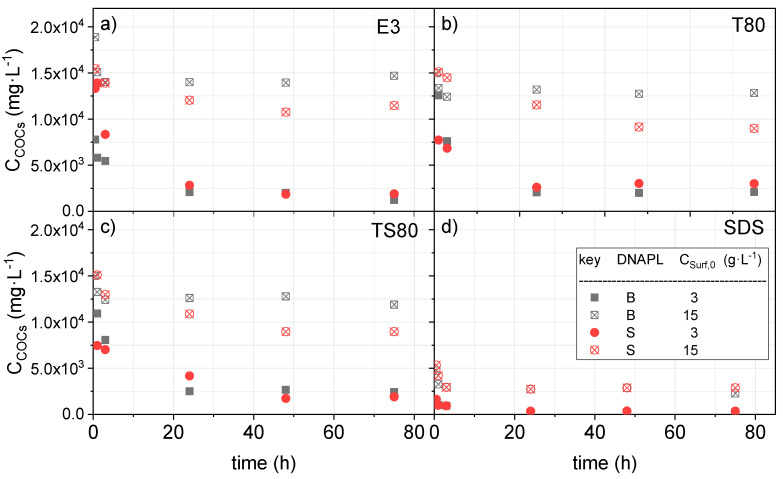
Evolution of chlorinated organic compounds (COCs) concentration in aqueous phase with (**a**) E3 at 3 and 15 g·L^−1^; (**b**) T80 at 3 and 15 g·L^−1^; (**c**) TS80 at 3 and 15 g·L^−1^; (**d**) sodium dodecyl sulfate (SDS) at 3 and 15 g·L^−1^ and pH = 7.

As explained in the experimental part, for each experiment in Table S3, a vial was sacrificed at different settling times, and the amount of COCs in the aqueous phase was measured. The profiles of COCs in solution with the settling time is shown in Figure 2. As can be seen, although the clay gradually precipitated during the settling of the COC, the concentration in the aqueous phase remained constant 24 h after the agitation had stopped and the equilibrium was achieved.

#### 3.2.1. Distribution of Solubilized COCs

As the DNAPL samples used in this research are a complex mixture of COCs, a study has been conducted to see if the surfactants selectively dissolve some compounds in the mixture. The molar distribution of COCs in the DNAPL sample is summarized in Table S2. The percentage of each solubilized COC in the aqueous phase is calculated as follows:

*y(%mole) = 100·[(C_j,AQ_/MW_j_)/(∑ C_j,AQ_/MW_j_)]_eq_*, (3)

where *C_j,AQ_* is the concentration of *j* in the aqueous phase, mg·L^−1^ and *MW_j_* are the molecular weight of *j.* In mg·mmol^−1^, COC isomers have been lumped together, so *j* refers to CB, the sum of DCBs, TCBs, TetraCBs, PCB, PentaCXs, HexaCXs, HCHs, and HeptaCHs isomers.

**Figure 3:**
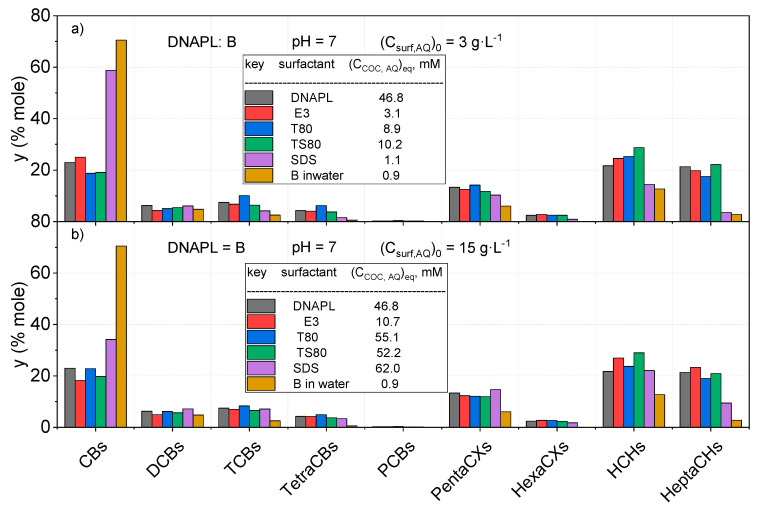
Molar distribution (%) of COCs in the initial DNAPL: B as a sum of isomers and COC distribution in the aqueous phase using an initial surfactant concentration of (**a**) *(C_surf,AQ_)_0_ =* 3 g·L^−1^ (**b**) *(C_surf,AQ_)_0_ =* 15 g·L^−1^ at pH = 7. The distribution of solubilized COCs in the aqueous phase saturated in DNAPL (without surfactant) is given as B in water.

**Figure 4:**
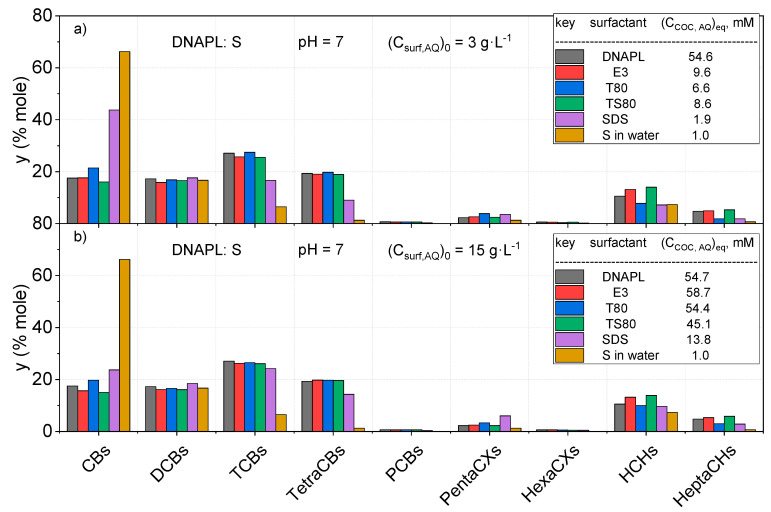
Molar distribution (%) of COCs in the initial DNAPL: S as the sum of isomers and COC distribution in the aqueous phase using an initial surfactant concentration of (**a**) *(C_surf,AQ_)_0_ = 3* g·L^−1^ (**b**) (C_surf,AQ_)_0_ = 15 g·L^−1^ at pH = 7. The distribution of solubilized COCs in water is also provided as DNAPL saturated in water.

The molar percentages of solubilized COCs (calculated by Equation (3)) under equilibrium conditions at two different initial surfactant concentrations (3 g·L^−1^ and 15 g·L^−1^) at neutral pH are shown in Figure 3 and Figure 4, for DNAPL: B and S, respectively. As can be seen, the higher the surfactant concentration, the higher the sum of solubilized COCs.

Moreover, the distribution of COCs in the aqueous phase without surfactant and DNAPL are also included. As can be seen, the distribution of COCs solubilized by nonionic surfactants is quite similar to the initial distribution of COCs in the organic phase (the latter is shown in Table S2), for both DNAPL: B (Figure 3) and S (Figure 4). Differences are lower than 15%, regardless of the initial surfactant concentration in the aqueous phase. This fact can be explained assuming that the organic phase is trapped in the micellar cores and the aqueous phase is emulsified. Therefore, for the nonionic surfactants tested, the complex mixture forming the DNAPL can be joined together as a single compound.

On the contrary, the solubilization of COCs without surfactant presents a different distribution, following the partitioning of these compounds in the aqueous phase reported elsewhere [6,39]. The distribution of COCs in the aqueous phase without surfactants is quite different from their distribution in the organic phase, where chlorobenzene is the most abundant dissolved COC.

On the other hand, COC distribution obtained with SDS at a low surfactant concentration is more similar to that found in the aqueous phase without surfactant, as can be seen in Figure 3 and Figure 4. However, the higher the SDS concentration, the more similar the distribution of dissolved COCs to that found in the organic phase, although some selectivity is still noticed.

The total COCs concentrations in the aqueous phase from the solubility experiments are shown in the legends of Figure 3 and Figure 4.

The effect of the addition of alkali on solubilized COC distribution in the presence of surfactants under equilibrium conditions was analyzed. COC distribution in the aqueous phase obtained at initial surfactant concentrations of 3 and 15 g·L^−1^ is summarized in Figures S6 and S7 for DNAPL: B and S, respectively. For comparison purposes, COC distribution at neutral pH and the total moles of dissolved COCs at neutral and alkaline pHs are also shown in these figures. The concentration of COCs in the aqueous phase is also summarized in the captions of the figures mentioned above.

As can be seen in Figures S6 and S7, for a given surfactant concentration, the total moles of dissolved COCs are similar at both pHs, but their distribution changes. PentaCX, HexaCX, HCH, and HeptaCH isomers are not detected in the aqueous emulsions under alkaline conditions while TCBs and TetraCB molar percentages have remarkably increased under these conditions. It has been reported elsewhere [39] that under alkali conditions, HCH and PentaCX isomers in the aqueous phase produce TCB and HeptaCHs compounds and HexaCXs are transformed into TetraCBs. Dehydrochlorination reactions and selectivity concerning TCB and TetraCB isomers obtained are shown in Figures S8 and S9, following that reported by Lorenzo et al. [39]. Therefore, the alkaline conditions produced the transformation of some compounds in a solubilized fraction of DNAPL inside the micelles generated by the surfactant due to alkaline conditions; however, similar molar total concentration of COCs in solution is obtained at neutral and alkaline pHs. Moreover, the TCBs and TetraCBs produced under alkaline conditions are less toxic than the precursors’ organic compounds (HCHs and HeptaCHs, respectively) [47,48,49,50].

#### 3.2.2. Partition Equilibria of Solubilized COCs

From the results shown in Table S5, it is clear that the concentration of solubilized COCs depends on the concentration of the surfactant added. In addition, it has been found that for a nonionic surfactant, the complex mixture of compounds in DNAPL can be joined together as a single solubilized compound.

The partition of COCs and equilibrium conditions can be modeled employing the MSR (or WSR) parameters defined in Equation (4).

*MSR = (C_COCs,AQ_)_eq_/(C_surf,AQ_/MW_surf_)_eq_*; *WSR = (∑C_j,AQ_)_eq_/(C_surf,AQ_)_eq_*, (4)

where *MW_surf_* is the molecular weight of the surfactant summarized in Table S1 (but for E3 that is unknown). The sum of COCs dissolved has been called C_COCs,AQ_, in mmol·L^−1^ defined as

*(C_COCs,AQ_)_eq_ = ∑(Cj_,AQ_/MW_j_)*. (5)

**Figure 5:**
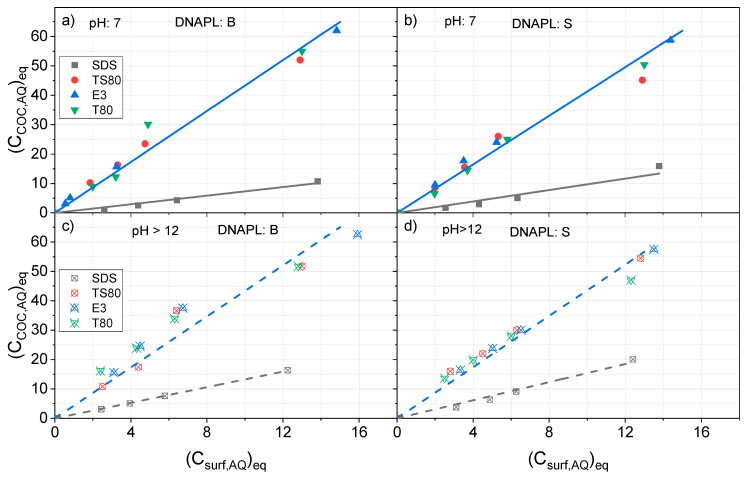
Aqueous phase solubility of COCs in mmol*_COCs_·*L^−1^ versus the concentration of surfactant in the aqueous phase in g*_surf_·*L^−1^ after reaching equilibrium at pH = 7 using (**a**) B, (**b**) S, and at pH > 12 using (**c**) B and (**d**) S.

In Figure 5, the concentration of solubilized COCs calculated by (5), in mmol·L^−1^, vs. the concentration of surfactant in the aqueous phase under equilibrium conditions, calculated by Equation (S2) in the Supplementary Information), in g L^−1^, has been plotted for both pHs and DNAPLs studied. As can be seen in Figure 5a,c and Figure 5b,d, similar plots are obtained for nonionic surfactants at both pHs. It is in concordance with the assumption that the DNAPL is mobilized as an organic phase in the micellar core and the reaction of dehydrochlorination by NaOH takes place in this aqueous phase. Moreover, similar results are obtained regardless of the DNAPL used. The pH only affects the solubilization capacity of SDS. The higher the pH, the higher the COC concentration solubilized with SDS. The explanation for this is that SDS selectively solubilized the COCs as explained in the previous section.

Moreover, the higher the surfactant concentration in solution, the higher the solubilized COC concentration, meaning that an almost linear trend is found between both variables in the concentration range studied. On the other hand, lower solubilization is achieved with SDS in comparison with that obtained with nonionic surfactants, following that reported by Zhou et al. in previous research [51].

The slope of the plots in Figure 5 can be related to the MSR or MWR values obtained in Equation (4), which are summarized in Table 2. Nonionic surfactant data at both pHs and for both DNAPLs were joined together. The statistical significance of linear regression parameters obtained is summarized in Table S6.

The SMR values obtained were in line with those reported by Pei et al. for the solubilization of DCBs using different surfactants [52].

In the linear regressions, the intercept corresponds to the solubilized DNAPL in the absence of the surfactant: 0.9 mmol·L^−1^ and 1.0 mmol·L^−1^ for B and S, respectively, following previous results [39].

## 4. Conclusions

The surfactants studied here (three nonionic surfactants (E3, TS80, and T80) and one anionic surfactant (SDS)) significantly improved the solubilization of a complex mixture of chlorinated organic compounds contained in real DNAPL present in two landfills polluted with liquid organic wastes from lindane production. This improvement was remarkably greater for all nonionic surfactants tested, finding similar values of molar solubilization ratios (MSR) regardless of the pH used. Moreover, a significant partition of the surfactants was found between the organic and the aqueous phases. The nonionic surfactant presented a higher affinity for the organic phase at neutral pH. For each DNAPL, the composition of COCs in the micellar core was similar to the initial composition of the organic phase for the three nonionic surfactants tested. In contrast, the anionic surfactant selectively solubilized the most polar compounds in DNAPL. Moreover, under strong alkaline conditions, dehydrochlorination of some COCs trapped in the micelles was noticed obtaining an emulsion with less toxic COC. These findings are relevant in the design of the surfactant-enhanced remediation process of the site.

## Figures and Tables

**Table 1:** Parameters estimated by fitting experimental data in Figure 1a,b to the Langmuir isotherm in Equation (1) and experimental data in Figure 1c,d to the linear partitioning model in Equation (2). The statistical significance of parameters is summarized in Table S4.

	**pH = 7**			**pH = 7**		
	DNAPL: B			DNAPL: S		
Surf.	*C_s,ORG_* g*_surf_·*g^−1^*_ORG_*	*K_S_* L·g^−1^*_surf_*	*K_L_* g*_surf,ORG_·*L·g^−1^*_surf·_*g^−1^*_ORG_*	*C_s,ORG_* g*_surf_·*g^−1^*_ORG_*	*K_S_* L·g^−1^*_surf_*	*K_L_* g*_surf,ORG_·*L·g^−1^*_surf·_*g^−1^*_ORG_*
SDS	0.093	0.143	0.013	0.093	0.159	0.015
T80 and TS80	0.387	0.155	0.059	0.354	0.097	0.034
E3	0.362	2.179	0.789	0.415	0.126	0.052
	**pH > 12**			**pH > 12**		
	*C_s,ORG_* g*_surf_·*g^−1^*_ORG_*	*K_S_* L·g^−1^*_surf_*	*K_L_* g*_surf,ORG_·*L·g^−1^*_surf·_*g^−1^*_ORG_*	*C_s,ORG_* g*_surf_·*g^−1^*_ORG_*	*K_S_* L·g^−1^*_surf_*	*K_L_* g*_surf,ORG_·*L·g^−1^*_surf·_*g^−1^*_ORG_*
All			0.020			0.016

**Table 2:** Molar solubilization ratios (MSR) and mass solubilization ratio (WSR) values calculated from the slopes in Figure 5 for nonionic surfactants and SDS *.

**Nonionic Surfactants pH = 7 and >12, DNAPL: B and S**			
	WSR mg*_COCs_*·g^−1^*_surf_*	MSR MW_S_ mmol*_COCs_*·g^−1^*_surf_*	MSR mmol*_COCs_*·mmol^−1^*_surf_*
T80			5.66
TS80	1005	4.33	3.11
E3			-
**Anionic surfactant: SDS surfactant**			
	WSR mg*_COCs_*·g^−1^*_surf_*	MSR MW_S_ mmol*_COCs_*·g^−1^*_surf_*	MSR mmol*_COCs_*·mmol^−1^*_surf_*
pH = 7 DNAPL: B	162	0.7	0.43
pH = 7 DNAPL: S	186	0.97	0.68
pH > 12 DNAPL: B	307	1.32	0.8
pH > 12 DNAPL: S	295	1.54	0.94

* The statistical significance of linear regression parameters obtained is summarized in Table S6.

## Supplementary Materials

The following are available online at https://www.mdpi.com/1660-4601/17/12/4494/s1, Table S1: Surfactant tested in DNAPL solubilization and main properties. Table S2: Mole fraction of COCs from B and S DNAPLs samples used. Table S3: Experimental conditions of the solubility tests carried out. wORGo = 400 mg, Vaq = 0.02 L, pH = 7 and >12 (7 g L^−1^ NaOH); DNAPL: B and S. Table S4: Parameters and statically significance obtain to fit Equations (4) and (5) to the data in Figure 1 at pH = 7 and pH > 12 for both DNAPLs, B and S. Table S5: Concentrations of surfactants and COCs in aqueous phase at neutral and alkaline pH and equilibrium state. Table S6: Parameters and statically significance obtain to fit Equation (9) to the data in Figure 4 at pH = 7 and pH > 12 for both DNAPLs, B and S. Figure S1: Left: organic phase S; right: organic phase B adding E3 at several concentrations (initial surfactant concentration from the left to the right: 3, 5, 7.5, and 15 g L^−1^) and pH = 7. Top: Appearance before agitation. Center: Appearance after agitation (5 h). Bottom: Appearance after 75 h of settling. Figure S2: Left: organic phase S; right: organic phase B adding T80 at several concentrations (initial surfactant concentration from the left to the right: 3, 5, 7.5, and 15 g L^−1^) and pH = 7. Top: Appearance before agitation. Center: Appearance after agitation (5 h). Bottom: Appearance after 75 h of settling. Figure S3: Left: organic phase S; right: organic phase B adding TS80 at several concentrations (initial surfactant concentration from the left to the right: 3, 5, 7.5, and 15 g·L^−1^) and pH = 7. Top: Appearance before agitation. Center: Appearance after agitation (5 h). Bottom: Appearance after 75 h of settling. Figure S4: Left: organic phase S; right: organic phase B adding SDS at several concentrations (initial surfactant concentration from the left to the right: 3, 5, 7.5, and 15 g L^−1^) and pH = 7. Top: Appearance before agitation. Center: Appearance after agitation (5 h). Bottom: Appearance after 75 h of settling. Figure S5: Appearance of emulsion at 75 h of settling after alkali addition: from the top to the bottom: E3, T80, TS80, and SDS. Left: Results with DNAPL from S, right: Results with B. Initial surfactant concentration from the left to the right: 3, 5, 7.5, and 15 g·L^−1^. Figure S6: Molar distribution (%) of COCs in the initial DNAPL: B as sum of isomers and COCs distribution in aqueous phase using a surfactant initial concentration of (a) *C_surf,AQ0_* = 3 g·L^−1^ (b) C_surf,AQ0_ = 15 g·L^−1^ at pH > 12. Figure S7: Molar distribution (%) of COCs in the initial DNAPL: S as sum of isomers and COCs distribution in aqueous phase using a surfactant initial concentration of (a) *C_surf,AQ0_* = 3 *g·L*^−1^ (b) *C_surf,AQ0_* = 15 g·L^−1^ at pH > 12. Figure S8: Reaction of HCH and PentaCX isomers in DNPLS to TCBs under alkali conditions adapted from (Lorenzo et al., 2020). Figure S9. Reaction of HeptaCH and HexaCX isomers in DNPLS to TetraCBs under alkali conditions. Adapted from (Lorenzo et al., 2020).
