# Supplementary material for: Surfactant-Enhanced Solubilization of Chlorinated Organic Compounds Contained in DNAPL from Lindane Waste: Effect of Surfactant Type and pH
Source: Int J Environ Res Public Health. 2020 Jun 23;17(12):4494. doi: 10.3390/ijerph17124494 (PMC7345101; doi:10.3390/ijerph17124494)
Supplement: Supplementary file 1 [file ijerph-17-04494-s001.pdf]

Surfactant-enhanced solubilization of chlorinated organic compounds contained in DNAPL from lindane waste: Effect of surfactant type and pH

Supplementary Material

Raul García-Cervilla[a], Arturo Romero[b], Aurora Santos[c] and David Lorenzo[d]*

Chemical and Materials Engineering Department. University Complutense of Madrid. Spain.

Keywords: DNAPL, Surfactants, Partition, Lindane wastes, Chlorinated Organic Compounds.

Table S1. Surfactant tested in DNAPL solubilization and main properties.

Surfactant	Purchased to	Aspect of solution	Molecular Formula	Molecular weight	CMC measured (mg L^{-1})	CMC literature (mg L^{-1})	$FR_{TOC,surf}$ *	HLB
E-Mulse®3	EthicalChem	slightly orange, transparent	Not available	unknown	80	Not available	0.58	Not available
Tween®80	Sigma-Aldrich	slightly orange, transparent	$C_{64}H_{124}O_{26}$	1308	20	13-15 [1]	0.60	15 [1]
Span®80	Sigma-Aldrich	cloudy white	$C_{24}H_{44}O_{6}$	404	10	7 [2]	0.69	4.3 [3]
Tween®80 (35%) - Span®80 (65%)	Mixture	cloudy white		718	11		0.60	Not available
SDS	Sigma-Aldrich	Transparent	$C_{12}H_{25}NaO_{4}S$	608	1800	1586 [1]	0.50	40 [1]

*$FR_{TOC,surf}$ $(g\ g^{-1})$= mass fraction of carbon per mass of surfactant as the response factor obtained from the calibration curves of pure surfactants solutions using TOC analysis

Table S2. Mole fraction of COCs from B and S DNAPLs samples used.

**Acronym**	**Name**	**CAS**	**MW**	$FR_{TOC,j}{}^{*}$	**B**	**S**
CB	Chlorobenzene	108-90-7	112	0.64	0.230	0.175
1,3 DCB	1,3-Dichlorobenzene	541-73-1	146	0.49	0.003	0.007
1,4 DCB	1,4-Dichlorobenzene	106-46-7	146	0.49	0.032	0.095
1,2 DCB	1,2-Dichlorobenzene	95-50-1	146	0.49	0.027	0.070
1,3,5 TCB	1,3,5-Trichlorobenzene	108-70-3	180	0.40	0.001	0.004
1,2,4 TCB	1,2,4-Trichlorobenzene	120-82-1	180	0.40	0.069	0.224
1,2,3 TCB	1,2,3-Trichlorobenzene	87-61-6	180	0.40	0.005	0.043
TetraCB (1,2,4,5+1,2,3,5)	1,2,4,5-Tetrachlorobenzene 1,2,3,5-Tetrachlorobenzene	95-94-3/ 634-90-2	214	0.34	0.020	0.087
TetraCB (1,2,3,4)	1,2,3,4-Tetrachlorobenzene	634-66-2	214	0.34	0.023	0.106
γ–PentaCX	γ-Pentachlorocyclohexene	342631-17-8	252	0.29	0.067	0.012
PentaCB	1,2,3,4,5-Pentachlorobenzene	608-93-5	248	0.29	0.002	0.007
δ-PentaCX	δ-Pentachlorocyclohexene	643-15-2	252	0.29	0.027	0.009
θ-PentaCX	θ-Pentachlorocyclohexene	319-94-8	252	0.29	0.006	0.001
HexaCX-a	Hexachlorocyclohexene	1890-41-1	289	0.25	0.010	0.003
β-PentaCX	β-Pentachlorocyclohexene	319-94-8	252	0.29	0.010	0.001
η-Penta CX	η-Pentachlorocyclohexene	54083-24-8	252	0.29	0.023	0.000
HexaCX-b	Hexachlorocyclohexene	1890-41-1	289	0.25	0.008	0.000
HexaCX-c	Hexachlorocyclohexene	1890-41-1	289	0.25	0.006	0.003
α-HCH	α-Hexachlorocyclohexane	319-84-6	291	0.25	0.035	0.020
HexaCX-d	Hexachlorocyclohexene	1890-41-1	289	0.25	0.001	0.000
β-HCH	β-Hexachlorocyclohexane	319-85-7	291	0.25	0.000	0.000
γ-HCH	γ-Hexachlorocyclohexane	58-89-9	291	0.25	0.099	0.068
HeptaCH-1	Heptachlorocyclohexane	707-55-1	322	0.22	0.142	0.028
δ-HCH	δ-Hexachlorocyclohexane	319-86-8	291	0.25	0.073	0.012
ε-HCH	ε-Hexachlorocyclohexane	6108-10-7	291	0.25	0.010	0.005
HeptaCH-2	Heptachlorocyclohexane	707-55-1	322	0.22	0.050	0.014
HeptaCH-3	Heptachlorocyclohexane	707-55-1	322	0.22	0.021	0.006
				Molecular Weight DNAPL	232	191

$*$ $FR_{TOC,surf}$ $(g\ g^{-1})$= mass fraction of organic carbon per mass of j COC.

Table S3. Experimental conditions of the solubility tests carried out. w_{ORG_0} = 400 mg V_{aq} = 0.02 L, pH = 7 and > 12 (7 g L-1 NaOH)*;* DNAPL: B and S

**Surfactant**	**$(C_{surf,AQ})_0$ (g·L^{-1})**
E3	0, 3.5, 5.7, 8.5, 17
SDS	0, 3, 5, 7.5, 15
T80	0, 3, 5, 7.5, 15
TS80	0, 3, 5, 7.5, 15

Table S4. Parameters and statically significance obtain to fit Eq. 4 and Eq.5 to the data in Figure 1 at pH=7 and pH> 12 for both DNAPLs, B and S.

		**B pH=7**			**S pH=7**		
**Surfactant**	**Parameter**	**Value**	**Standard Error**	**R^2**	**Value**	**Standard Error**	**R^2**
SDS	$C_{s,or}$ $g_{surf} \cdot g_{DNAPL}^{-1}$	0.093	0.027	0.98	0.093	0.024	0.91
	K $g_{aq} \cdot g_{surf}^{-1}$	0.144	0.087		0.159	0.090	
TS80 and T80	$C_{s,or}$ $g_{surf} \cdot g_{DNAPL}^{-1}$	0.387	0.075	0.91	0.354	0.085	0.99
	K $g_{aq} \cdot g_{surf}^{-1}$	0.155	0.062		0.097	0.041	
E3	$C_{s,or}$ $g_{surf} \cdot g_{DNAPL}^{-1}$	0.362	0.023	0.97	0.415	0.153	0.98
	K $g_{aq} \cdot g_{surf}^{-1}$	2.179	0.609		0.126	0.091	
		**B pH>12**			**S pH>12**		
		**Value**	**Standard Error**	**R^2**	**Value**	**Standard Error**	**R^2**
All		0.020	0.001	0.97	0.016	0.001	0.93

Table S5. Concentrations of surfactants and COCs in aqueous phase at neutral and alkaline pH and equilibrium state.

Organic phase	pH = 7							
	E3		SDS		T80		TS80	
	$(C_{surf,AQ})_{eq}$ (g L^{-1})	$(C_{COC,AQ})_{eq}$ (mmol L^{-1})	$(C_{surf,AQ})_{eq}$ (g L^{-1})	$(C_{COC,AQ})_{eq}$ (mmol L^{-1})	$(C_{surf,AQ})_{eq}$ (g L^{-1})	$(C_{COC,AQ})_{eq}$ (mmol L^{-1})	$(C_{surf,AQ})_{eq}$ (g L^{-1})	$(C_{COC,AQ})_{eq}$ (mmol L^{-1})
B	0.5	3.1	2.6	1.2	2.0	8.9	1.9	10.2
	0.8	5.1	4.4	2.5	3.2	12.3	3.3	16.3
	3.2	15.7	6.4	4.3	4.9	30.2	4.7	23.5
	14.8	62.0	13.8	10.7	12.0	55.1	12.9	52.0
S	2.0	9.55	2.5	2.0	2.0	6.6	2.0	8.6
	3.5	17.7	4.3	3.6	3.7	14.5	3.6	15.46
	5.2	24.0	6.3	5.3	5.8	25.0	5.3	26.0
	14.4	58.7	13.8	12.9	13.0	54.4	12.9	45.1
Organic phase	pH > 12							
	E3		SDS		T80		TS80	
	$(C_{surf,AQ})_{eq}$ (g L^{-1})	$(C_{COC,AQ})_{eq}$ (mmol L^{-1})	$(C_{surf,AQ})_{eq}$ (g L^{-1})	$(C_{COC,AQ})_{eq}$ (mmol L^{-1})	$(C_{surf,AQ})_{eq}$ (g L^{-1})	$(C_{COC,AQ})_{eq}$ (mmol L^{-1})	$(C_{surf,AQ})_{eq}$ (g L^{-1})	$(C_{COC,AQ})_{eq}$ (mmol L^{-1})
B	3.1	15.5	2.4	3.1	2.4	16.2	2.5	15.5
	4.5	24.6	3.9	5.1	4.3	23.9	4.4	24.6
	6.7	37.5	5.8	7.6	6.3	33.9	6.4	36.6
	15.9	62.5	12.3	16.3	12.8	51.7	13.0	51.7
S	3.3	24.1	3.1	3.8	2.5	13.6	2.8	16.0
	5.0	32.6	4.9	6.4	4.0	19.9	4.5	21.9
	6.5	38.1	6.3	9.1	6.0	28.0	6.3	30.1
	13.5	63.0	12.4	20.0	12.3	47.0	12.0	54.4

Table S6. Parameters and statically significance obtain to fit Eq. 9 to the data in Figure 4 at pH=7 and pH>12 for both DNAPLs, B and S.

	B pH=7			S pH=7		
	**Value**	**Standard Error**	**R^2**	**Value**	**Standard Error**	**R^2**
SDS	0.70	0.03	0.99	1.03	0.08	0.97
Nonionic surfactants	4.33	0.22	0.97	4.05	0.21	0.96
	B pH>12			S pH>12		
	**Value**	**Standard Error**	**R^2**	**Value**	**Standard Error**	**R^2**
SDS	1.32	0.01	0.99	1.54	0.06	0.99
Nonionic surfactants	4.34	0.18	0.97	4.36	0.20	0.97

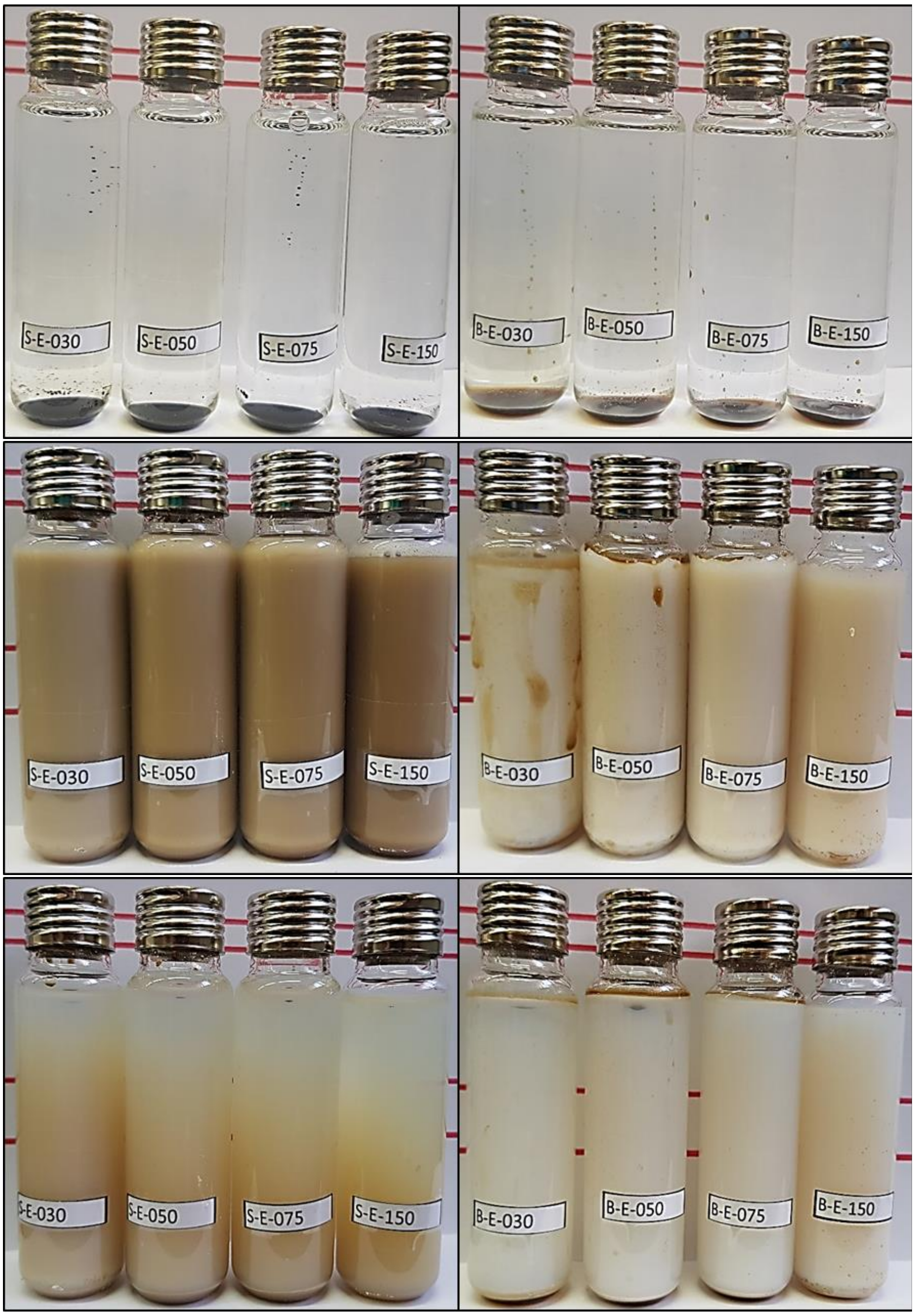

Figure S1. Left: organic phase S; Right: organic phase B adding E3 at several concentrations (Initial surfactant concentration from the left to the right: 3, 5, 7.5 and 15 g L^{-1}) and pH=7. Top: Appearance before agitation. Centrum: Appearance after agitation (5h). Bottom: Appearance after 75 h of settling.

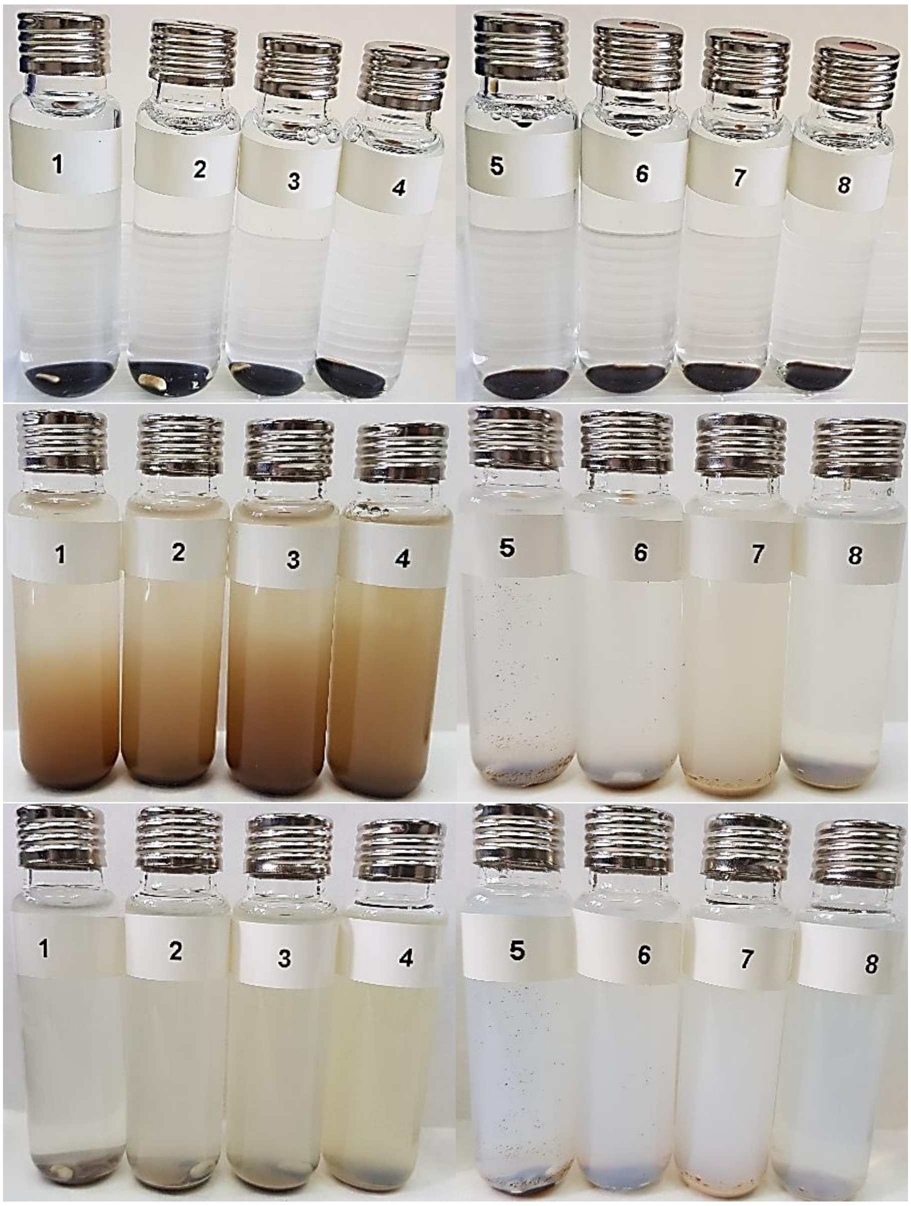

Figure S2. Left: organic phase S; Right: organic phase B adding T80 at several concentrations (Initial surfactant concentration from the left to the right: 3, 5, 7.5 and 15 g L^{-1}) and pH=7. Top: Appearance before agitation. Centrum: Appearance after agitation (5h). Bottom: Appearance after 75 h of settling.

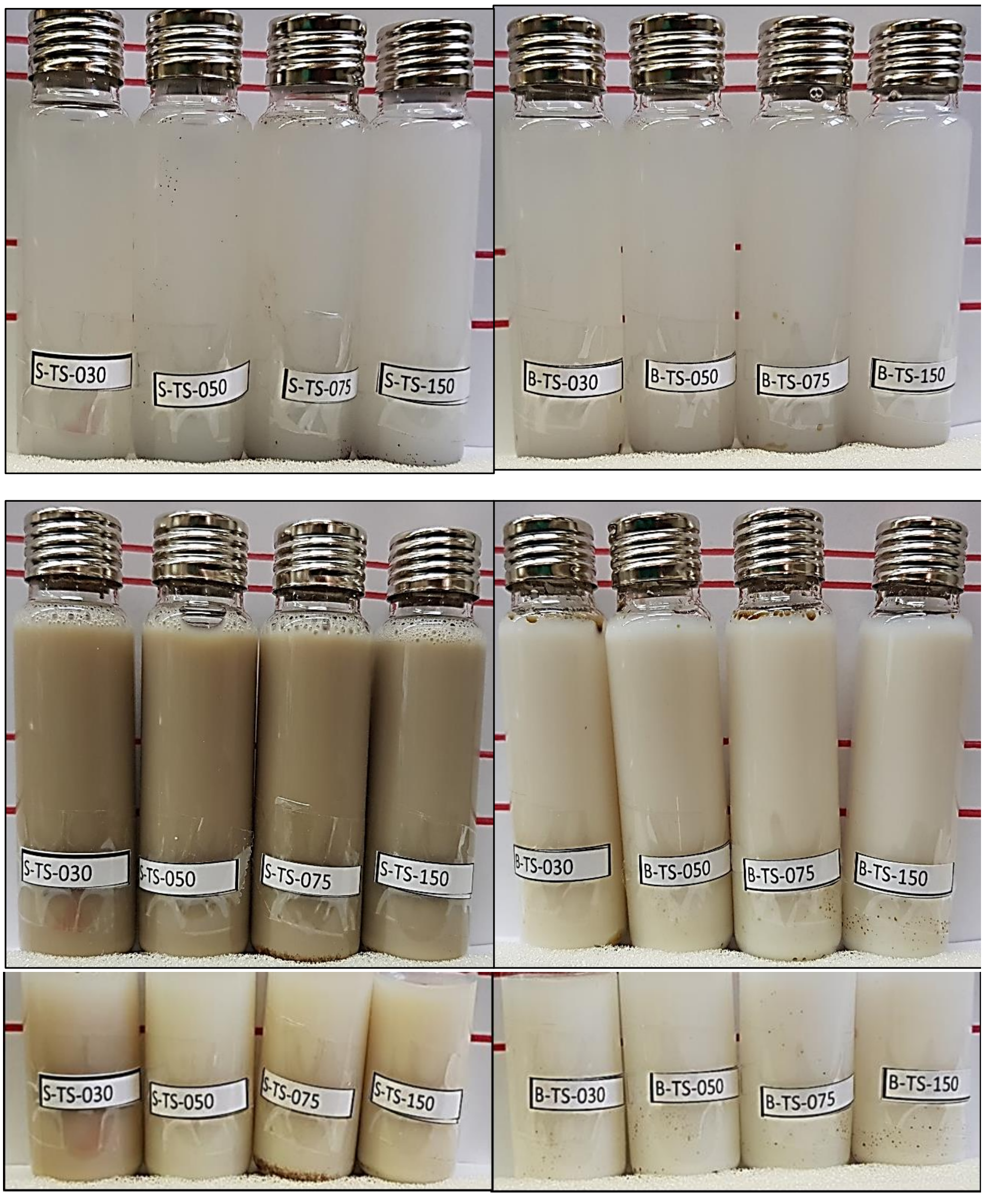

Figure S3. Left: organic phase S; Right: organic phase B adding TS80 at several concentrations (Initial surfactant concentration from the left to the right: 3, 5, 7.5 and 15 g L^{-1}) and pH=7. Top: Appearance before agitation. Centrum: Appearance after agitation (5h). Bottom: Appearance after 75 h of settling.

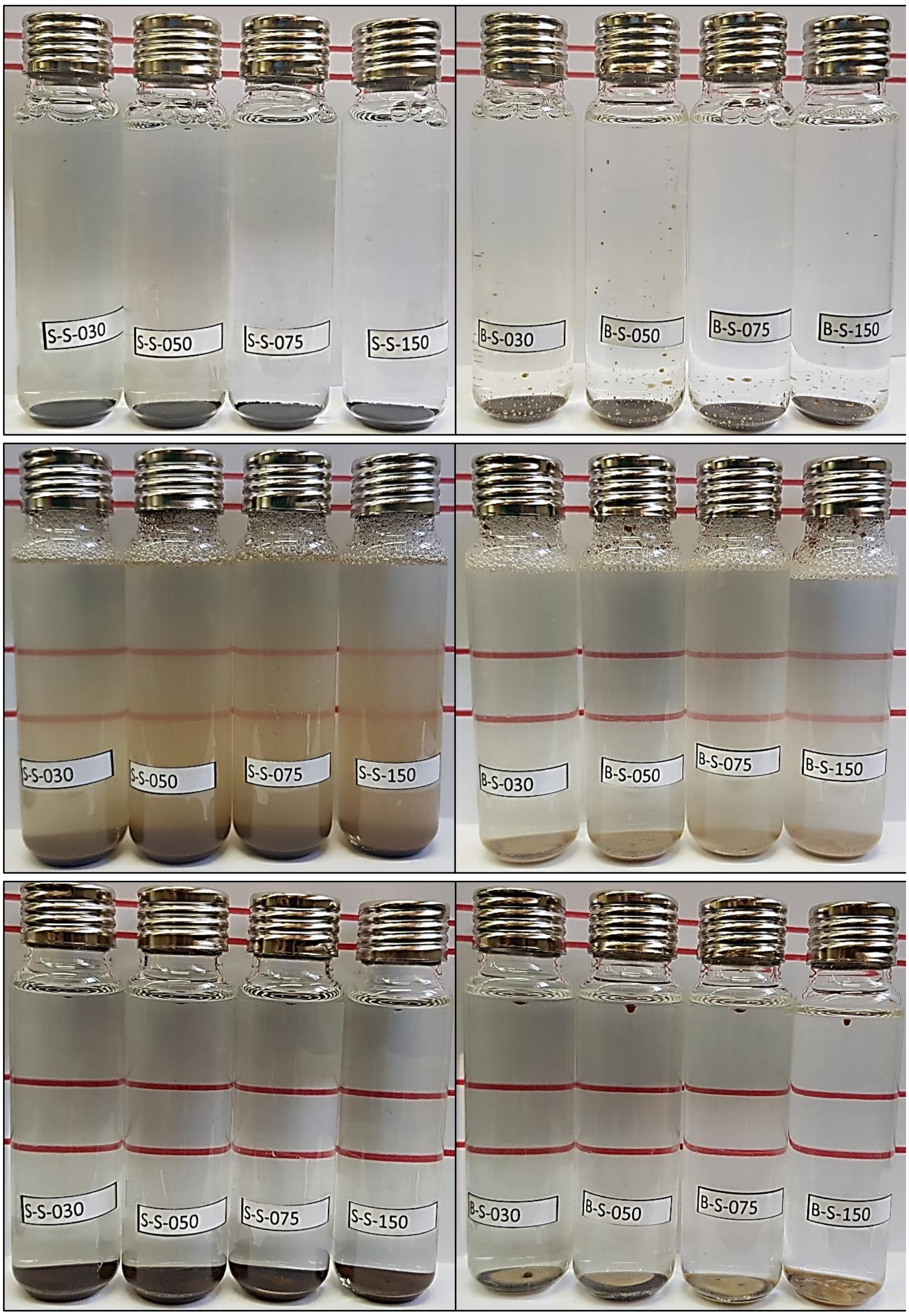

Figure S4. Left: organic phase S; Right: organic phase B adding SDS at several concentrations (Initial surfactant concentration from the left to the right: 3, 5, 7.5 and 15 g L^{-1}) and pH=7. Top: Appearance before agitation. Centrum: Appearance after agitation (5h). Bottom: Appearance after 75 h of settling.

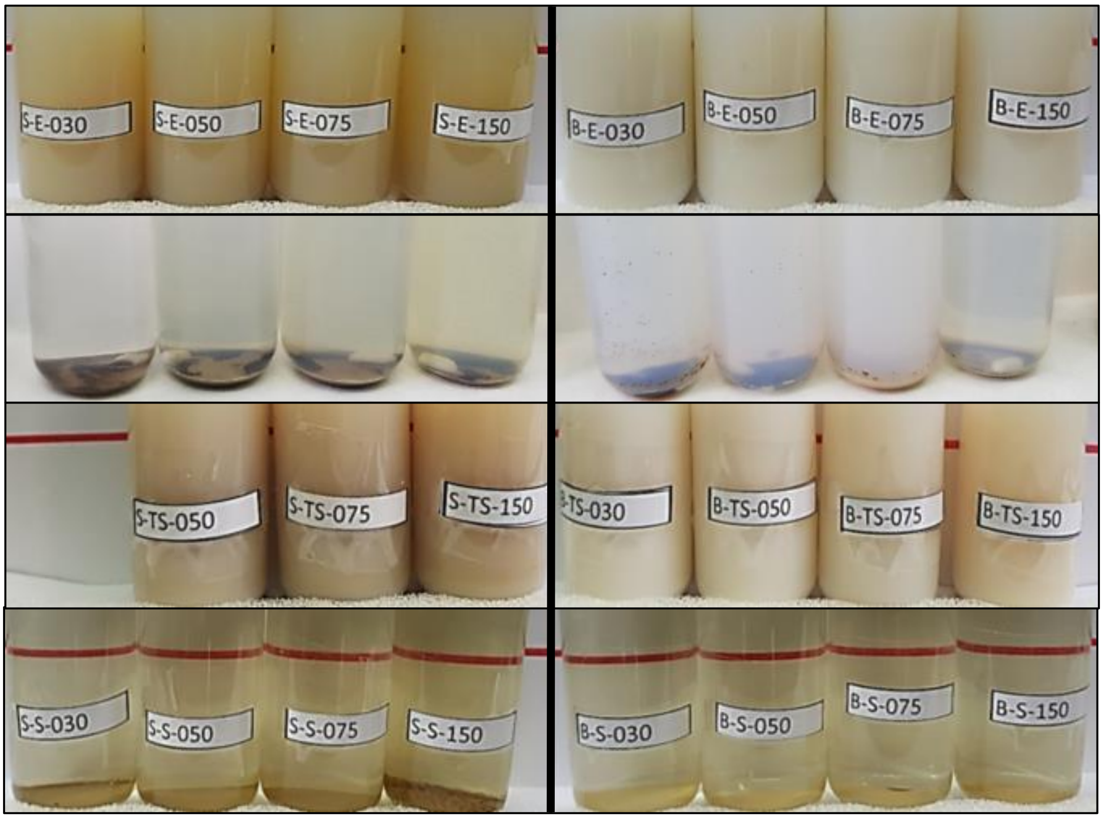

Figure S5. Appearance of emulsion at 75 h of settling after alkali addition: From the top to the bottom: E3, T80, TS80 and SDS. Left: Results with DNAPL from S, Right: Results with B. Initial surfactant concentration from the left to the right: 3, 5, 7.5 and 15 g L^{-1}.

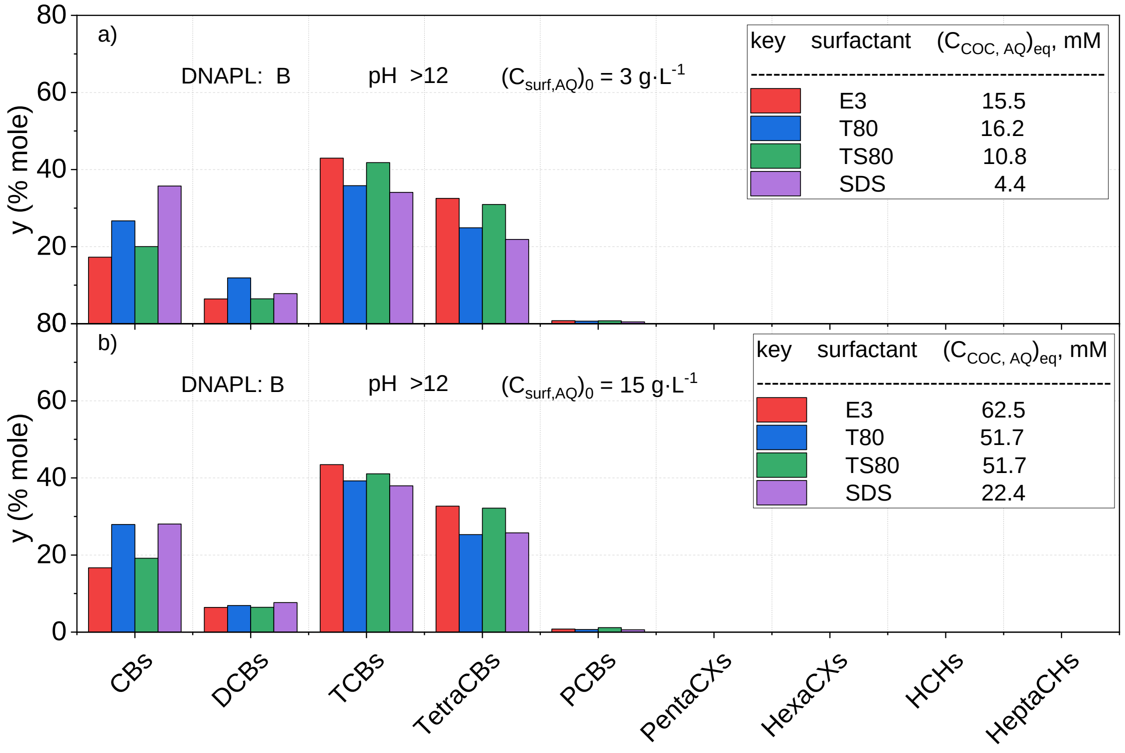

Figure S6. Molar distribution (%) of COCs in the initial DNAPL: B as sum of isomers and COCs distribution in aqueous phase using a surfactant initial concentration of a) $\left(C_{surf,AQ}\right)_0 = 3g\ L^{-1}$ b) $\left(C_{surf,AQ}\right)_0 = 15\ g\ L^{-1}$ at pH >12.

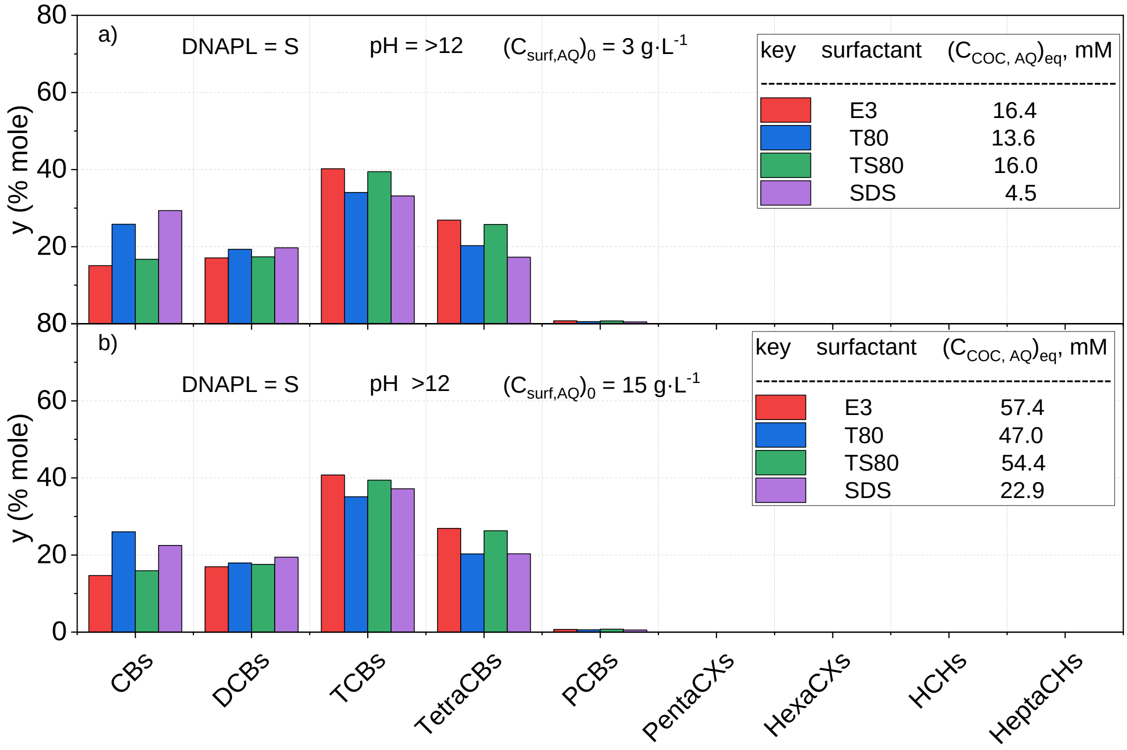

Figure S7. Molar distribution (%) of COCs in the initial DNAPL: S as sum of isomers and COCs distribution in aqueous phase using a surfactant initial concentration of a) $\left(C_{surf,AQ}\right)_0 = 3g\ L^{-1}$ b) $\left(C_{surf,AQ}\right)_0 = 15\ g\ L^{-1}$ at pH >12.

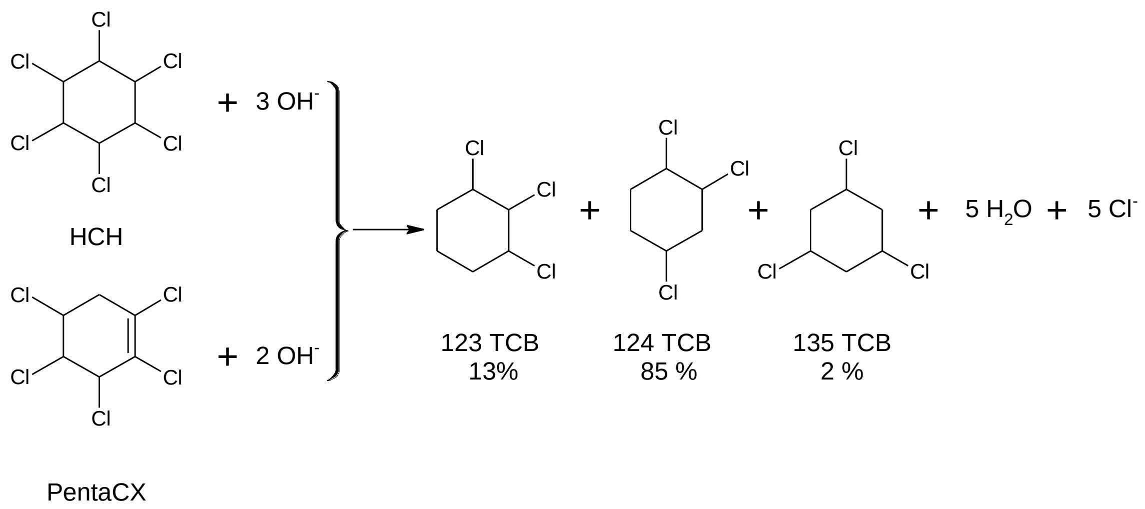

Figure S8. Reaction of HCH and PentaCX isomers in DNPLS to TCBs under alkali conditions adapted from [4].

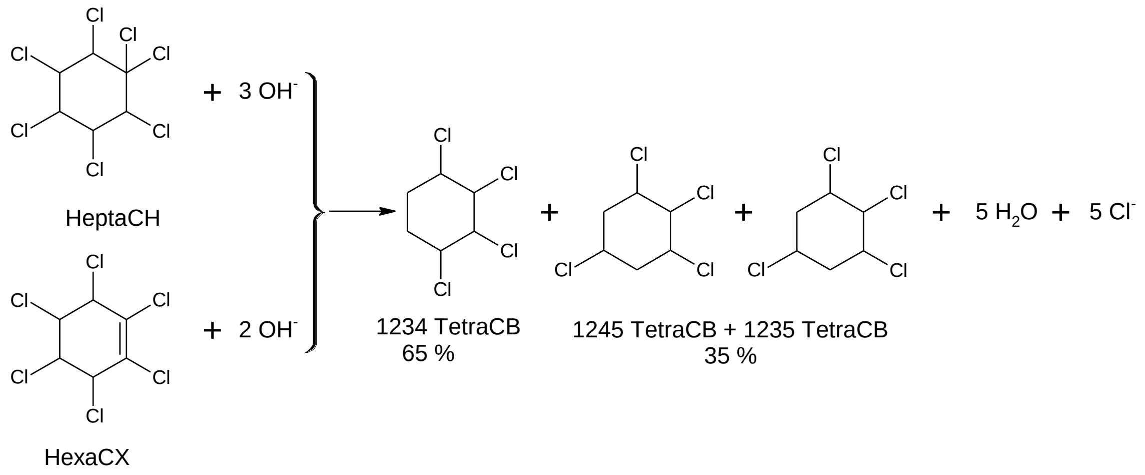

Figure S9.. Reaction of HeptaCH and HexaCX isomers in DNPLS to TetraCBs under alkali conditions. Adapted from [4].

Surfactatn and DNAPL mass balance under equilibrium donfitions:

The absorbed amount of surfactant in the organic phase under equilibrium conditions, $(C_{surf,ORG})_{eq}$, is calculated by Eq. (1) in $g_{surf}\cdot g^{-1}_{org}$. The concentration of surfactant under equilibrium conditions in the aqueous phase$(C_{surf,AQ})_{eq}$, is calculated by Eq. (2) in $g_{surf}\cdot L^{-1}$. The mass of organic phase in the aqueous volume when equilibrium is achieved, $(w_{ORG})_{EQ}$, is calculated with Eq.(3) in g_{ORG}.

$$(C_{surf,ORG})_{eq}=[(C_{surf,AQ})_0-(C_{surf,AQ})_{eq}]\cdot V_{aq}/(w_{ORG})_{EQ} \quad (1)$$

$$(C_{surf,AQ})_{eq}=\{(C_{TOC,AQ})_{eq}-\sum[(C_{j,AQ})_{eq}\cdot FR_{TOC,j}]/(FR_{TOC,surf}\cdot\cdot 1000) \quad (2)$$

$$(w_{ORG})_0=w_{DNAPL,0}-V_{aq}\cdot\sum (C_{j,AQ})_{eq} \quad (3)$$

$(C_{surf,AQ})_0$ is the initial concentration of surfactant in the aqueous solution in $g\cdot L^{-1}$, $(C_{TOC,AQ})_{eq}$ is the concentration of TOC measured in the aqueous phase under equilibrium conditions, $mg\cdot L^{-1}$, $(C_{j,AQ})_{eq}$ is the concentration of the organic pollutant j in the aqueous phase under equilibrium conditions, $mg\cdot L^{-1}$, V_{aq} is the volume of the aqueous phase, L, and $FR_{TOC,surf}$ and $FR_{TOC,j}$ is the mass of carbon in the mass of each surfactant or each j compound, respectively, which are summarized in Table S1.

1. Liang, C.; Hsieh, C.-L. Evaluation of surfactant flushing for remediating EDC-tar contamination. *J. Contam. Hydro.l* **2015**, *177*, 158-166, doi:10.1016/j.jconhyd.2015.04.006.

2. Peltonen, L.J.; Yliruusi, J. Surface pressure, hysteresis, interfacial tension, and CMC of four sorbitan monoesters at water-air, water-hexane, and hexane-air interfaces. *J. Colloid. Interf. Sci.* **2000**, *227*, 1-6, doi:10.1006/jcis.2000.6810.

3. Farooq, A.; Shafaghat, H.; Jae, J.; Jung, S.-C.; Park, Y.-K. Enhanced stability of bio-oil and diesel fuel emulsion using Span 80 and Tween 60 emulsifiers. *Journal of Environmental Management* **2019**, *231*, 694-700, doi:10.1016/j.jenvman.2018.10.098.

4. Lorenzo, D.; García-Cervilla, R.; Romero, A.; Santos, A. Partitioning of chlorinated organic compounds from dense non-aqueous phase liquids and contaminated soils from lindane production wastes to the aqueous phase. *Chemosphere* **2020**, *239*, 124798, doi:https://doi.org/10.1016/j.chemosphere.2019.124798.
